# Total hip arthroplasty for posttraumatic osteoarthritis secondary to acetabular fracture: An evidence based on 1,284 patients from 1970 to 2018

**DOI:** 10.3389/fsurg.2022.953976

**Published:** 2022-11-10

**Authors:** Qiling Yuan, Xinyi Wang, Yongsong Cai, Mingyi Yang, Haishi Zheng, Xiaoming Zhao, Hongyun Ma, Peng Xu

**Affiliations:** ^1^ Department of Joint Surgery, Xi’an Honghui Hospital, Xi’an Jiaotong University, Xi’an, China; ^2^ Department of Rehabilitation, Shaanxi Provincial Rehabilitation Hospital, Xi’an, China; ^3^ Department of Orthopedics of the First Affiliated Hospital, Medical School, Xi’an Jiaotong University, Xi’an, China

**Keywords:** total hip arthroplasty, acetabular fracture, posttraumatic osteoarthritis, systematic review, meta-analysis

## Abstract

**Background:**

Posttraumatic osteoarthritis (PTOA) can be a crippling sequela of acetabular fracture (AF), and total hip arthroplasty (THA) is often necessary to alleviate the clinical progression of symptoms. The purpose of this study was to summarize the existing clinical evidence concerning the surgical management of AF with THA through meta-analyses.

**Methods:**

Databases were searched for articles published between 1995 and January 2022 that contained the keywords “acetabular,” “fracture,” “arthroplasty,” and “osteoarthritis.” Our study was registered in PROSPERO under number CRD42022314997.

**Results:**

We screened 3,125 studies and included data from 31 studies with 1,284 patients. The median patient age at the time of THA was 52 years and ranged from 19 to 94 years. The pooled overall survival rate was 88% [86%–90%, 95% confidence interval (CI)] and could reach 83% at ≥15-year follow-up. For the Harris Hip Score, we pooled 22 studies with an overall mean difference of 43.25 (40.40–46.10, 95% CI; *P* < 0.001), indicating a large clinical effect. The pooled complications (incidence rates) across studies were: heterotopic ossification (22.53%), implant dislocation (4.66%), implant infection (3.44%), and iatrogenic nerve injury (1.07%).

**Conclusion:**

THA in patients with PTOA following AF leads to significant improvement in symptoms and function at ≥15-year follow-up. Survival rates of implants free from re-operation or revision after THA decreased with follow-up time and could still reach 83% at ≥15-year follow-up. THA might be an effective therapeutic method for patients with PTOA due to AF.

## Introduction

Acetabular fracture (AF) is a complex, high-energy injury with a poor prognosis despite treatment (1). Although open reduction and internal fixation (ORIF) is associated with a more favorable functional outcome for AFs (2), posttraumatic osteoarthritis (PTOA) still occurs at an incidence of approximately 13%–44% (3). PTOA can occur for many reasons, such as articular cartilage injury, intra-articular screws, non-anatomic reduction, or avascular necrosis (AVN) of the femoral head (4).

PTOA is a serious sequela of AF, and relief of clinical symptoms can often only be achieved by total hip arthroplasty (THA) (4). Giannoudis et al. (3) conducted a review and found that 9% of AF patients required conversion to THA on average 2 years after the initial surgery. However, due to local tissue changes, including scar tissue development, bone density changes, and infection, THA after AF is challenging and can result in increased operative time, blood loss, and/or poor acetabular placement (4). Sermon et al. found that the conversion rate of AF patients to THA was up to 22% after initial treatment failure (5). Patients with a history of AF who undergo THA are at risk for multiple complications, some of which are often the primary causes of surgical revision (4), such as heterotopic ossification (HO), surgical site infection, and prosthesis loosening. Several studies have shown that THA in PTOA secondary to AF is worse than THA in patients with non-traumatic primary osteoarthritis (POA) across a variety of indicators, including survival and complication rates (6).

THA has been recommended as an effective means of restoring normal hip function and is often used to restore joint function after treatment of AF has failed (6). Studies on THA for PTOA patients following AF have increased in recent years (7–9). However, these studies have various shortcomings, such as small sample sizes, unreasonable study design, different durations of follow-up, and inconsistent reported results. Therefore, we hope to summarize the existing clinical evidence through a systematic review to find more evidence to help improve the long-term efficacy of AFs and to reduce complications.

The purpose of this study was to summarize the existing clinical evidence concerning the surgical management of acetabular injuries with THA through meta-analyses. The primary aim of our study was to pool the survival rates to get the overall survival rate and the survival rates at different follow-up times. The secondary aim was to evaluate the outcomes of posttreatment with that of pretreatment in AF patients with THA. The last aim was to compare outcomes of delayed THA following AFs to outcomes of AFs treated with primary THA and non-traumatic primary THA.

## Methods

This review was priorly registered in PROSPERO with a number of CRD42022314997, and conducted according to Preferred Reporting Items for Systematic Reviews and Meta-Analyses guideline (10).

### Study selection criteria

#### Types of studies

Studies of any sample size were included for analysis in our review. We considered only studies with full-text manuscripts published in peer-reviewed journals in English with available follow-up data. Animal experiments, letters, and conference abstracts were excluded.

#### Types of participants and intervention

We included studies with patients of all ages who had undergone delayed THA for the treatment of PTOA following ORIF or conservative treatment (CST) for traumatic AF. Delayed THA was defined as an interval of >3 weeks between the AF and THA. We excluded patients with developmental hip dysplasia, pathologic fractures, periprosthetic fractures, stress fractures, and co-existing femur fractures. Studies with >30% of patients lost to follow-up were excluded to reduce the risk of attrition bias.

#### Types of outcomes

The primary outcome was the overall implant survival rate, defined as the rate of implants remaining free of re-operation or revision after THA. The secondary outcomes were clinical function measurements, such as the Harris Hip Score (HHS), Oxford Hip Score (OHS), Merle d'Aubigne Score (MDA), and Western Ontario and McMaster Universities Arthritis Index (WOMAC). Other factors of interest were as follows: patient demographics, the interval between AF and THA, operative characteristics (operative time, blood loss volume, and length of hospital stay), and complications (HO, implant dislocation, implant loosening, periprosthetic fracture, and infection).

### Search methods for study identification

We searched the PubMed, EMBASE, MEDLINE, and Cochrane Library databases from 1995 to January 15, 2022, for articles containing the keywords “acetabular,” “fracture,” “arthroplasty,” and “posttraumatic arthritis.” The detailed search strategy is presented in the Supplementary Material. Our database searches on January 15, 2022, yielded a total of 3,152 results.

Two independent reviewers (QLY and XYW) selected studies based on their titles and abstracts. Once a study was chosen, the full text was checked. The kappa-value statistic was used to measure agreement between the two reviewers. Disagreements were resolved by a third party (YSC).

### Data extraction, selection, and coding

The data were extracted from the studies using pilot-tested standardized data tables. The study details (author and publication year), district, number of hips, diagnoses, population characteristics (age, gender), the interval between fracture and THA, type of AFs, previous surgical treatments, follow-up times, complications, and operative data (operative time, blood loss, and hospital stay) were summarized in tables. Furthermore, the outcome measurements and implant survival rates were extracted, including HHS, OHS, MDA, and survival rate. Two independent reviewers (QLY, XYW) extracted the data. Discrepancies in data extraction results were resolved by a third, independent review author (YSC).

### Strategy for data synthesis

The data were grouped into continuous and dichotomous variables and pooled using a random effects model (the DerSimonian–Laird method for mean differences [MDs] and the Mantel–Haenszel method for risk ratios [RRs]). Heterogeneity between studies was evaluated using the *I*^2^ statistic of the *χ*^2^ test. A cutoff point of 50% and a *P* value <0.10 on the *χ*^2^ test indicated a significant degree of heterogeneity. Sensitivity analyses were performed to identify trials that disproportionately contributed to the observed heterogeneity. This was accomplished using jack-knife analysis, omitting each study one by one to assess its impact on the summary estimate. Meta-trim analysis was used to explore possible missing trials and to verify the robustness of the results after these trials were added. Subgroup analyses were performed according to the source of heterogeneity, where possible. Follow-up time was used as the primary variable for subgroup analyses. Publication bias was explored using a contour-enhanced funnel plot and Egger's test, where possible. All results are presented with 95% confidence intervals (CIs). All analyses were performed using STATA 14.0 software (StataCorp LP, College Station, TX).

## Results

### Literature search and demographic characteristics

**Figure 1:**
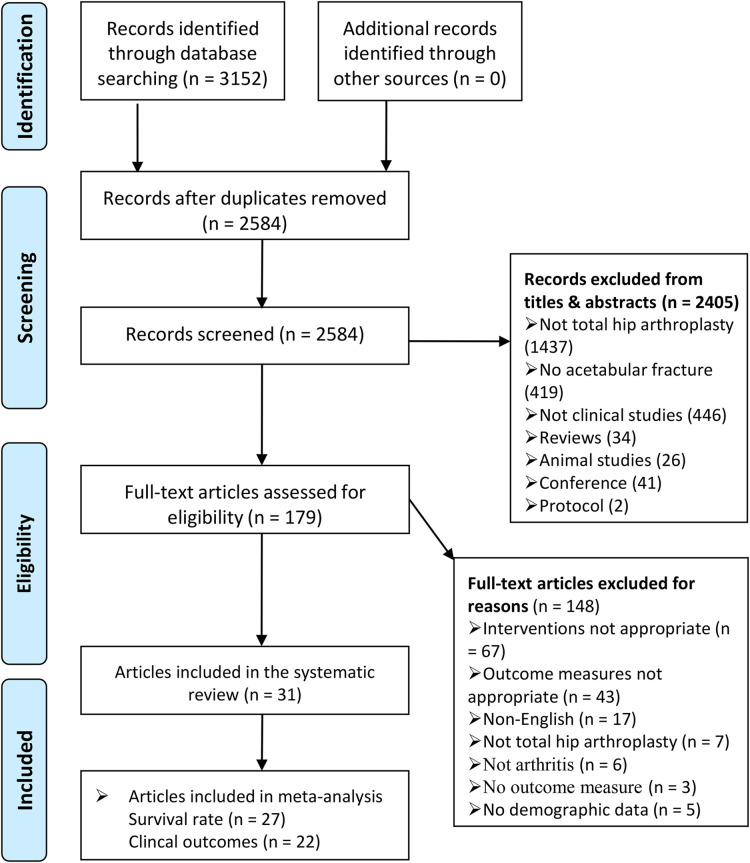
PRISMA flow chart.

**Figure 2:**
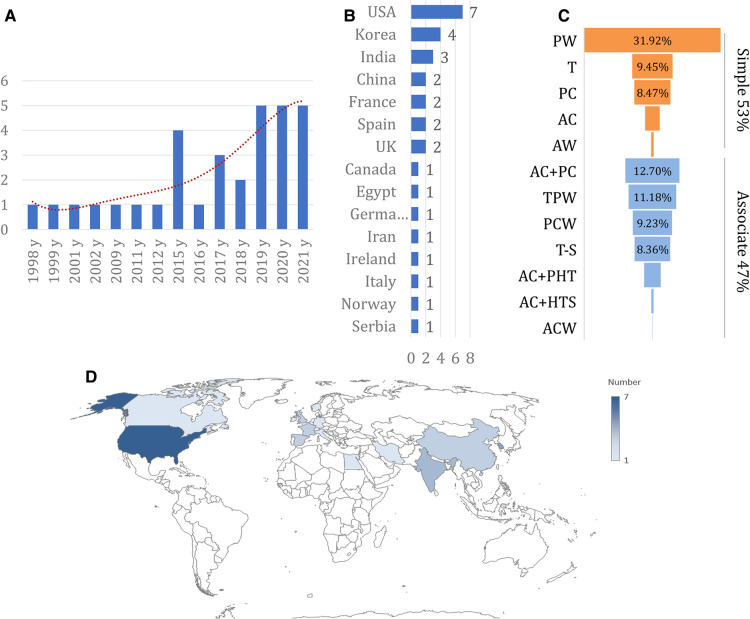
Basic characteristics of studies included. (**A**), number of included studies each year; (**B**), number of included studies in different countries; (**C**), the detail of fracture pattern; (**D**), the location of the included studies presented on world map.

Our search strategy identified 3,152 potentially eligible studies (Figure 1). A total of 568 duplicates were excluded, and 2,405 additional studies were also excluded based on their titles or abstracts. After full-text articles were assessed, 148 studies were excluded. Eventually, 31 studies (1,284 patients 1, 7, 9, 11–38); were included, with sample sizes ranging from 18 to 78 subjects. These studies were published between 1998 and 2021, and nearly half (15 studies 7, 9, 11–23); were published in the past 3 years (Figure 2A). Furthermore, 11 studies were conducted in Europe, 10 in Asia, 8 in North America, 1 in Africa (Figures 2B,D), 7 in the United States, 4 in South Korea, 3 in India, and 2 in China (Figure 2B). Twenty-three of the studies were retrospective and eight were prospective. The median patient age (based on the date of THA) across studies was 52 years (IQR 49.15 to 55.30) and ranged from 19 to 94 years of age. The proportion of females ranged from 5.13% to 53.33% (median 25.51%). The median follow-up time was 5.4 years (IQR 4.17 to 8.80), ranging from 7 days to 20 years. Tables 1, 2 and Supplementary Table S4 illustrate the main features of the included studies.

**Table 1:** Demographic characteristics of studies included.

Author	Year	District	No. of hips	Diagnosis	Age, range (mean ± SD) (year)	Female/Male (Female%)	BMI	Follow up (years)	Study year	Study design
Milenkovic (11)	2021	Serbia	23	PTOA 14; AVN 9	51.5 ± 13.8	5/18 (21.7%)	NA	1 week	2008–2018	RTP
Lucchini (7)	2021	Italy	68	PTOA 68	47.67 ± 11.6, (R 22–75)	8/60 (11.8%)	NA	11.8 ± 1.6, (R 10.2–17.7)	2000–2008	RTP
Kumar (12)	2021	India	18	PTOA 18	44.72 ± 11.99, (R 20–68)	4/14 (22.2%)	NA	2.3 ± 1.28, (R 1–5)	2015–2020	RTP
Gracia (13)	2021	France	60	PTOA 36; AVN 3	59 (R 20–94)	7/53 (12%)	NA	5 (R 2–11)	2004–2014	RTP
El-Bakoury (14)	2021	UK	40	PTOA 30; AVN 10	46.7 ± 16.1, (R 21–77)	9/31 (22.5%)	NA	4.17 (IQR 0.13–7.25)	2007–2019	RTP
Moon (15)	2020	Korea	37	PTOA 27; AVN 6; both 4	56.2 (R 24–81)	10/27 (27.0%)	NA	6.6 (R 2–12.1)	2002–2017	RTP
Min (16)	2020	Korea	39	PTOA 39	54.4 (R 29–81)	2/37 (5.4%)	NA	8.5 (R 5–15.7)	2001–2012	RTP
García-Rey (17)	2020	Spain	78	PTOA; AVN	56.9, (R 23–84)	30/48 (38.46%)	NA	11.7 (R 5–23)	1986–2012	RTP
Do (18)	2020	Korea	25	PTOA 22; AVN 3	58 (R 36–85)	6/19 (24%)	NA	4.17 (R 2–18.5)	2000–2016	RTP
Busch (19)	2020	Germany	67	PTOA; AVN	59 (R 25–87)	13/54 (19%)	NA	4.5 ± 1.95, (R 1.17–7.33)	2007–2012	RTP
Taheriazam (20)	2019	Iran	49	PTOA 49	NA	6/43 (12.24%)	NA	3.7, (R 2–5)	1998–2015	RTP
Sharma (9)	2019	India	47	PTOA 16; AVN 14; ACD 17	48.6 (R 25–83)	6/47 (12.77%)	NA	2.5 (R 1–5)	2007–2014	RTP
Lee (21)	2019	Korea	57	PTOA 57	52.5 (R 19–83)	26/31 (45.61%)	25.3	7.8 (R 4.67–13.5)	2003–2012	PRP
Giunta (22)	2019	France	27	NA	68.5 (R 57–84)	4/23 (14.81%)	NA	1.7 (R 1–7)	2010–2015	RTP
Dawson (23)	2019	Ireland	25	PTOA; AVN	53.8	8/17, (47.06%)	NA	2.3	2013–2017	RTP
Wang (24)	2018	China	33	PTOA 23; AVN 10	45.1 ± 9.3, (R 25–68)	12/21 (36.36%)	23.5	11.5 (R 8–17)	1997–2008	RTP
Salama (25)	2018	Egypt	21	PTOA 19; AVN 2	56.7 (R 29–75)	9/12 (42.86%)	NA	2.2 (R 2–3)	2011–2014	PRP
Scott (26)	2017	UK	49	PTOA 31; AVN 12; Others 6	57.4 (R 25–87)	16/33 (32.65%)	NA	9.1 (R 0.5–23)	1992–2016	PRP
Gavaskar (27)	2017	India	47	PTOA; AVN	48 ± 9	16/31 (34.01%)	28.5	7 ± 1.42	2006–2010	RTP
Clarke (28)	2017	Norway	52	PTOA; AVN	NA	17/35 (52%)	NA	7.3 (R 1–21)	1995–2014	PRP
Morison (32)	2016	Canada	74	PTOA 60; AVN 14	52 ± 12, 51 (R 25–75)	24/50 (32.43%)	NA	8 (R 2–23)	1987–2011	RTP
Yuan (29)	2015	United States	30	PTOA 30	45 (R 23–75)	9/21 (30%)	32	5 (R 2.1–10)	1999–2010	RTP
Roth (30)	2015	United States	66	PTOA 66	52 (R 19–80)	24/42 (36.36%)	NA	20 (R 3–40)	1970–1993	PRP
Chiu (33)	2015		56	PTOA; AVN	NA	NA	NA	10 (R 5–15)	1996–2010	RTP
Lizaur (34)	2012	Spain	24	PTOA 13; AVN 11	54 (R 28–77)	5/19 (20.83%)	27.1	8.4 ± 4.7, (R 5–15)	1992–2005	PRP
Zhang (35)	2011	China	55	PTOA; AVN	46.6 (R 22–65)	11/42 (20.75%)	25.7	5.3 (R 2.6–10.2)	1998–2007	RTP
Ranawat (1)	2009	United States	32	PTOA; AVN	52 (R 20–87)	9/23 (28.12%)	NA	4.7 (R 2–9.7)	1995–2003	RTP
Berry (36)	2002	United States	34	PTOA; AVN	49.7 (R 19–78)	NA	NA	11.93 (R 10–16)	1984–1990	PRP
Bellabarba (37)	2001	United States	30	PTOA 17; AVN 13	51 (R 26–86)	16/14 (53.33%)	NA	5.25 (R 2–11.7)	1984–1995	PRP
Huo (31)	1999	United States	21	PTOA; AVN	52 (R 23–78)	2/19 (9.52%)	32	5.4 (R 4–9)	1985–1993	RTP
Weber (38)	1998	United States	66	PTOA 66	52 (R 19–80)	24/42 (36.36%)	NA	9.6 (R 2–20)	1970–1993	RTP

AVN, avascular necrosis of the femoral head; BMI, body mass index; C, cemented; CHP, combined hip procedure (ORIF + THA); CoC, ceramic-on-ceramic bearings; CST, conservative treatment or non-surgical treatment; H, hybrid; NA, not applicable; ORIF, open reduction and internal fixation; OREF, open reduction and external fixation; PTOA, post-traumatic osteoarthritis; POA, primary osteoarthritis; PRP, prospective design; R, range; RTP, retrospective design; SD, standard deviation; THA, total hip arthroplasty; UC, uncemented.

**Table 2:** Clinical characteristics of studies included.

Author	Year	Type of THA	Interval between fracture and THA (years)	Previous surgical treatment	Type of previous surgery	Comparison	Type of endoprosthesis	Follow up (yrs)	Level of Evidence
Milenkovic (11)	2021	Delayed	4.28 (R 1–8)	YES 23	ORIF 23	NO	C 4, UC 15, H 4	1 week	IV
Lucchini (7)	2021	Delayed	11.9 (R 0.3–40)	YES 50; NO 18	ORIF 47, OREF 1, Unkown 2	ORIF 50 vs. CST 18	UC CoC 68	11.8 ± 1.6, (R 10.2–17.7)	IV
Kumar (12)	2021	Delayed	>1	YES 18	ORIF 18	NO	UC 14, H 4	2.3 ± 1.28, (R 1–5)	IV
Gracia (13)	2021	Delayed	2.42 (R 0.33–9.58)	YES 19; NO 20	ORIF 19	Acute vs. Delayed	C for acute 21; UC for delayed 39	5 (R 2–11)	III
El-Bakoury (14)	2021	Delayed	1.67 (R 0.25–12)	YES 25; NO 15	ORIF 30	ORIF 25 vs. CST 15	All cup and 29 stem for UC, 11 stem for C	4.17 (IQR 0.13–7.25)	IV
Moon (15)	2020	Delayed	4.83 (R 0.33–28)	YES 37	ORIF 37	ORIF vs. CST	UC, C	6.6 (R 2–12.1)	IV
Min (16)	2020	Delayed	NA	YES 27; NO 12	ORIF 27	ORIF 27 vs. CST 12	UC	8.5 (R 5–15.7)	IV
García-Rey (17)	2020	Delayed	5.79 (R 0.17–22.33)	YES 29; NO 49	ORIF 29	ORIF 29 vs. CST 49	UC 61, C 10, H 7	11.7 (R 5–23)	III
Do (18)	2020	Delayed	5.83 (R 0.58–17.75)	YES 25	ORIF 25	NO	UC 25	4.17 (R 2–18.5)	IV
Busch (19)	2020	Delayed	8.92 (R 0.08–42)	YES 67	ORIF	NO	NA	4.5 ± 1.95, (R 1.17–7.33)	IV
Taheriazam (20)	2019	Delayed	6.2 (R 3.2–17.1)	YES; NO	ORIF	NO	UC 47, C 2	3.7, (R 2–5)	IV
Sharma (9)	2019	Delayed	NA	YES 18; NO 29	ORIF	ORIF 18 vs. CST 29	UC 32, C 3, H 3	2.5 (R 1–5)	IV
Lee (21)	2019	Delayed	19 (R 0.16–60)	YES or NO	ORIF	PTOA 57 vs. POA 57	UC CoC	7.8 (R 4.67–13.5)	IV
Giunta (22)	2019	Delayed	NA	YES 6; NO 21	ORIF	ORIF 6 vs. CST 21	C 27	1.7 (R 1–7)	III
Dawson (23)	2019	Delayed	>4	YES 25	ORIF 25	NO	UC 14, C 10	2.3	IV
Wang (24)	2018	Delayed	4.83 (R 0.33–20)	YES 21; NO 12	ORIF 21	ORIF 21 vs. CST 12	UC 33 (CoC 21, MOP 12)	11.5 (R 8–17)	III
Salama (25)	2018	Delayed	NA	YES 17; NO 4	ORIF	ORIF 17 vs. CST 4	UC 21	2.2 (R 2–3)	III
Scott (26)	2017	Delayed	6.5 (R 0.1–25) yrs	YES 38; NO 11	ORIF 38	PTOA 49 vs. POA 98	C 49 (MOP 44, CoC 5)	9.1 (R 0.5–23)	III
Gavaskar (27)	2017	Delayed	2.19 ± 0.83	YES 27; NO 20	ORIF	ORIF 27 vs. CST 20	UC 47	7 ± 1.42	III
Clarke (28)	2017	Delayed	2.4 (R 0.1–14.1)	NA	NA	UC vs. C	UC 10, C 33, H 9	7.3 (R 1–21)	IV
Morison (32)	2016	Delayed	4 (R 1–24)	YES 58; NO 16	ORIF	ORIF 58 vs. CST 16	UC 70, H4	8 (R 2–23)	III
Yuan (29)	2015	Delayed	9 (R 0.33–42)	YES 30	ORIF 30	porous metal 30 vs. others 51	C porous metal 30	5 (R 2.1–10)	IV
Roth (30)	2015	Delayed	NA	YES 66	ORIF 66	UC vs. C	UC 20, C 44, H 2	20 (R 3–40)	IV
Chiu (33)	2015	Delayed	NA	YES; NO	ORIF	ORIF vs. CST	UC 56	10 (R 5–15)	III
Lizaur (34)	2012	Delayed	3 (R 0.42–14)	YES 9; NO 14	ORIF 9	PTOA 24 vs. POA 48	UC 24	8.4 ± 4.7, (R 5–15)	III
Zhang (35)	2011	Delayed	6.08 (R 0.58–30)	YES 30; NO 25	ORIF 30	ORIF 30 vs. CST 25	UC 47, C 7 (CoC 9)	5.3 (R 2.6–10.2)	III
Ranawat (1)	2009	Delayed	3 (R 0.08–18.92)	YES 24; NO 8	ORIF 24	ORIF 24 vs. CST 8	UC 32 (MOM 3, COP 10, MOP 19)	4.7 (R 2–9.7)	III
Berry (36)	2002	Delayed	NA	YES 15; NO 19	ORIF 15	ORIF 15 vs. CST 19	UC 34	11.93 (R 10–16)	IV
Bellabarba (37)	2001	Delayed	3.08 (R 0.67–37)	YES 15, NO 15	ORIF 15	ORIF 15 vs. CST 15; PTOA 30 vs. POA 184	UC 30	5.25 (R 2–11.7)	IV
Huo (31)	1999	Delayed	13.67 (R 0.67–40)	YES 7, NO 14	ORIF 7	ORIF 7 vs. CST 14	UC 21	5.4 (R 4–9)	IV
Weber (38)	1998	Delayed	NA	YES 66	ORIF 66	UC 20 vs. C 44	UC 20, C 44, H 2	9.6 (R 2–20)	IV

AVN, avascular necrosis of the femoral head; C, cemented; CHP, combined hip procedure (ORIF + THA); CoC, ceramic-on-ceramic bearings; CST, conservative treatment or non-surgical treatment; H, hybrid; NA, not applicable; ORIF, open reduction and internal fixation; OREF, open reduction and external fixation; PTOA, post-traumatic osteoarthritis; POA, primary osteoarthritis; R, range; THA, total hip arthroplasty; UC, uncemented.

### Perioperative variables

Patients were diagnosed with PTOA or PTOA plus AVN of the femoral head; however, no study reported the PTOA grade. The interval between fracture and THA was 4.83 years (IQR 4.07–6.02), ranging from 1.67 years to 19 years. The fracture type was reported for 1,000 patients (Supplementary Table S2). According to Letournel's classification system, 534 patients showed simple patterns and 466 showed associated patterns. The most common patterns were posterior wall (31.92%), anterior column plus posterior column (12.70%), transverse plus posterior wall (11.18%), and transverse (9.45%) (Figure 2C). In 28 studies, treatment of the fracture (i.e., ORIF or CST) was reported, and the proportion of patients undergoing ORIF ranged from 22.22% to 100%. Most endoprostheses were uncemented. Body mass index (BMI) was reported in seven studies (24, 27, 29, 31, 34, 35), with a median of 27.1 kg/m^2^. Ten studies (1, 21–25, 27, 30, 31, 34, 38) reported average operative times, with a median of 110 min (ranging from 81 to 188 min; Supplementary Table S3). Only seven studies (1, 21–24, 27, 31, 37, 38) reported average blood loss, with a median of 797.8 ml (ranging from 448 to 960 ml). Four studies (21–23, 31) reported average hospital stay, which ranged from 5 to 23.6 days.

### Survival rate of implant

**Figure 3:**
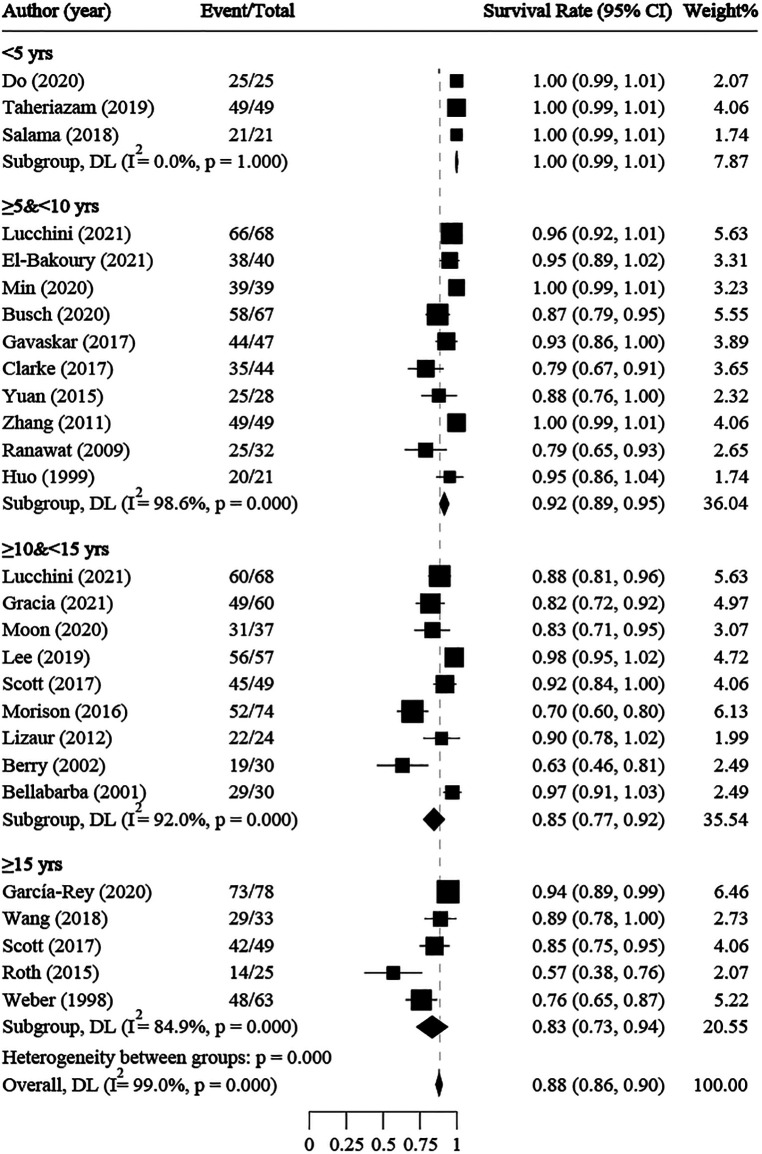
Overall and subgroup meta-analyses for implant survival rate.

The survival rate of implants without re-operation or revision after THA was reported in 27 studies, with a median of 90% and a range of 57% to 100%. The average follow-up time ranged from 1 to 20 years. Single-rate meta-analyses were conducted, and subgroup meta-analyses were performed by follow-up time. The overall survival rate was 88% (86%–90%, 95% CI) (Figure 3). The subgroup survival rates were 100% at <5 years, 92% (89%–95%) at ≥5 and <10 years, 85% (77%–92%) at ≥10 and <15 years, and 83% (73%–94%) at ≥15 years. Moreover, a significant negative correlation was found between survival rate and follow-up time (*R*^2^ = 0.9449, *y* = −0.058*x* + 1.045). Meta-regressions showed no statistically significant correlations between implant survival rate and age (coefficient = −0.0007, *P* = 0.948), publication year (coefficient = −0.0056, *P* = 0.996), simple AF rate (coefficient = 0.0172, *P* = 0.997), or AVN rate (coefficient = −0.1561, *P* = 0.975) (Supplementary Figure S1).

### Posttreatment vs. pretreatment

**Figure 4:**
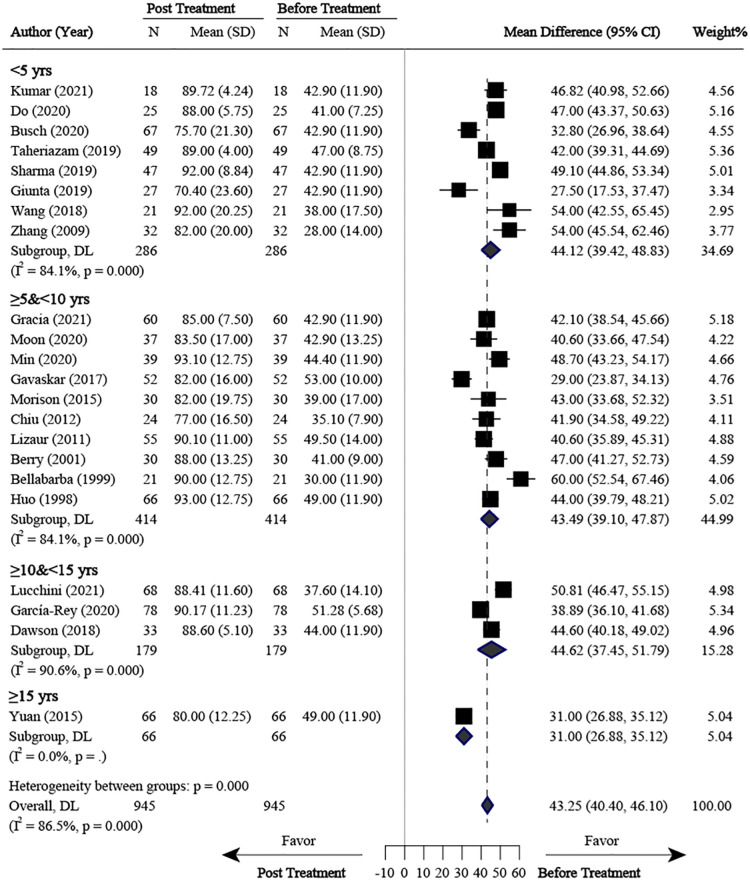
Meta-analysis for HHS comparison between posttreatment and pretreatment. HHS, harris hip score; SD, standard deviation.

**Figure 5:**
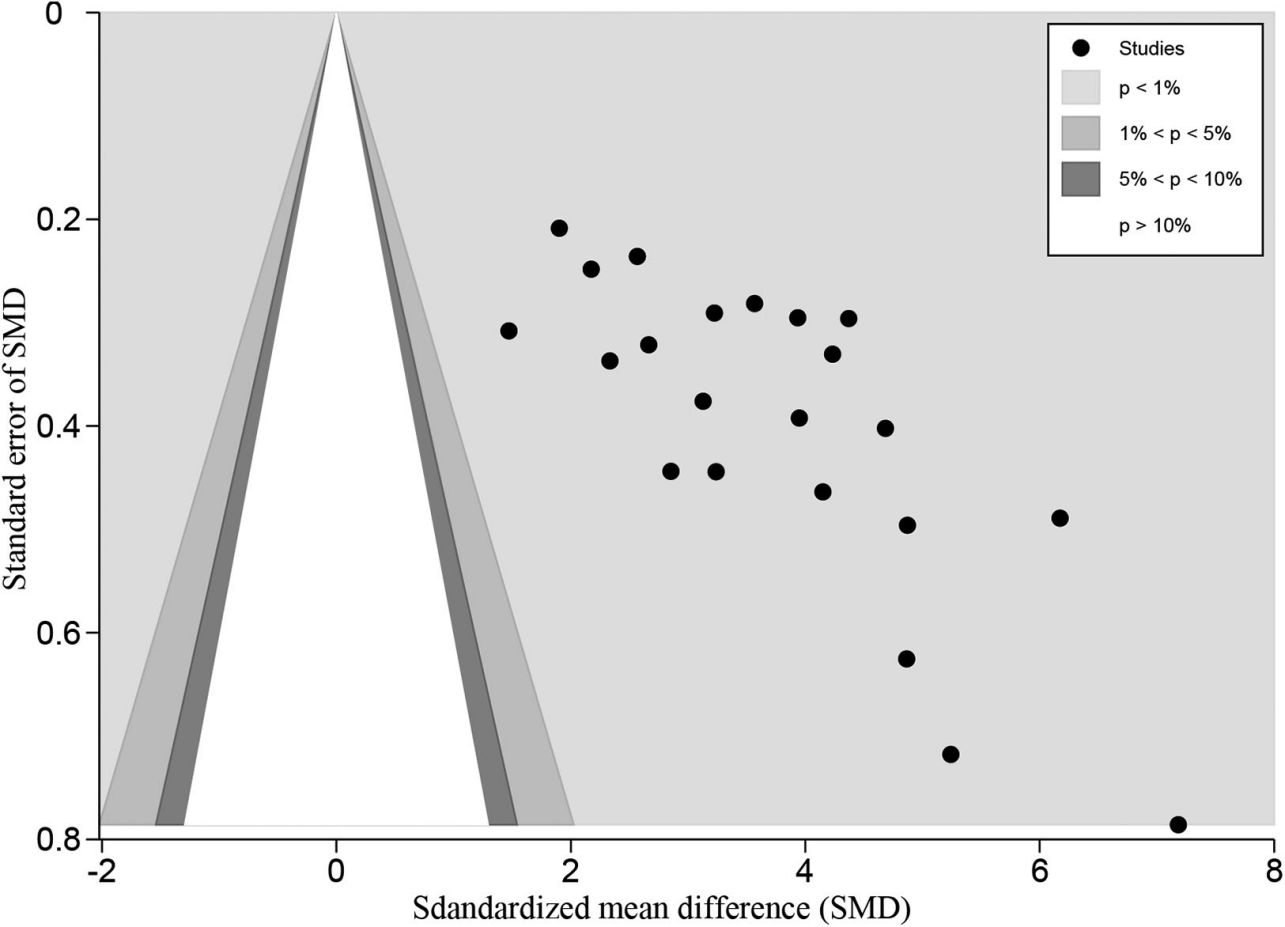
Confunnel plot for HHS comparison between posttreatment and pretreatment. HHS, harris hip score.

**Figure 6:**
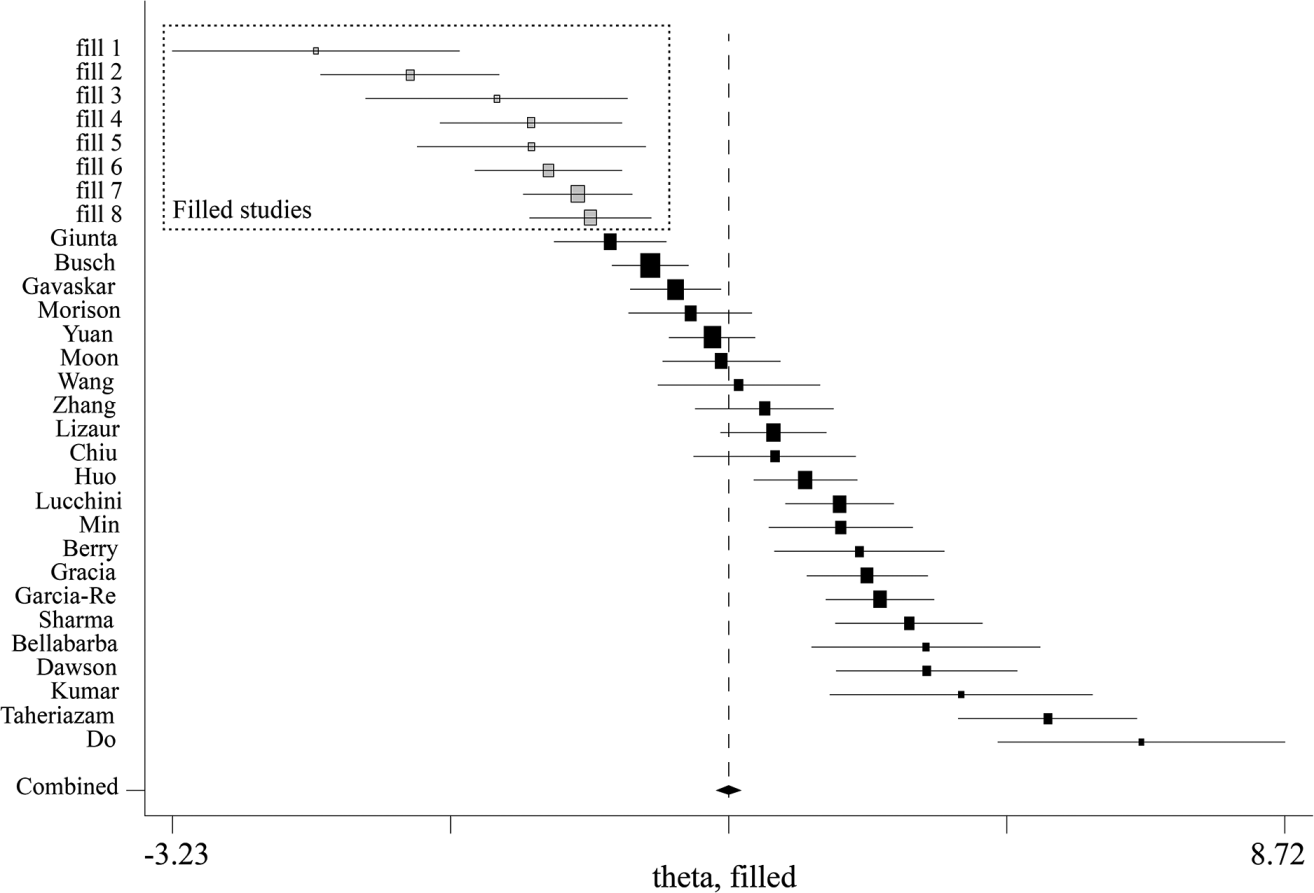
Trim and fill analysis for HHS comparison between posttreatment and pretreatment. HHS, harris hip score.

Comparison between posttreatment and pretreatment was conducted across the studies. The following clinical outcomes were reported: HHS, OHS, and MDA. For HHS, 22 studies (7, 9, 12, 13, 15–20, 22–24, 27, 29, 31–37) were identified and pooled with an overall MD of 43.25 (40.40–46.10; *P* < 0.001), indicating a large clinical effect (Figure 4). Although significant heterogeneity was found across these studies (*I*^2 ^= 86.5%, *P *< 0.001), jack-knife analysis showed that the results were robust. Publication bias was identified by confunnel plot (Figure 5) and Egger's test (coefficient = −1.08; *P* = 0.055). Nevertheless, we conducted a trim-and-fill analysis; eight studies were filled, which indicated that publication bias had a significant effect on the results (Figure 6). Subgroup analysis was conducted by follow-up time, and no significant differences were found between subgroups. Meta-regressions based on variables, such as age, the proportion of females, and the interval between fracture and THA were conducted, and no statistically significant correlation was observed between these variables and effect size (*P* > 0.05) (Supplementary Figure S2). For OHS, four studies (13, 14, 26, 27) with an MD of 25.22 were pooled, indicating a large effect (19.99–30.46; *P* < 0.001) (Supplementary Figure S3). Significant heterogeneity was found among these four studies (*I*^2 ^= 94.3%, *P* < 0.001). For MDA, two studies (11, 27) were pooled with an MD of 5.60 (4.69–6.52; *P* < 0.001), which indicated a large effect (Supplementary Figure S4). Significant heterogeneity was found between these two studies (*I*^2 ^= 84.8%, *P* = 0.010). An excellent and good rate based on HHS (HHS score ≥80) after THA was reported in 14 studies. The pooled results revealed that the overall excellent and good rate was 0.79 (0.70–0.87, 95% CI) (Supplementary Figure S5). Subgroup analysis according to follow-up time was not significantly different between subgroups (*P *> 0.05).

### Open reduction and internal fixation vs. conservative treatment

**Figure 7:**
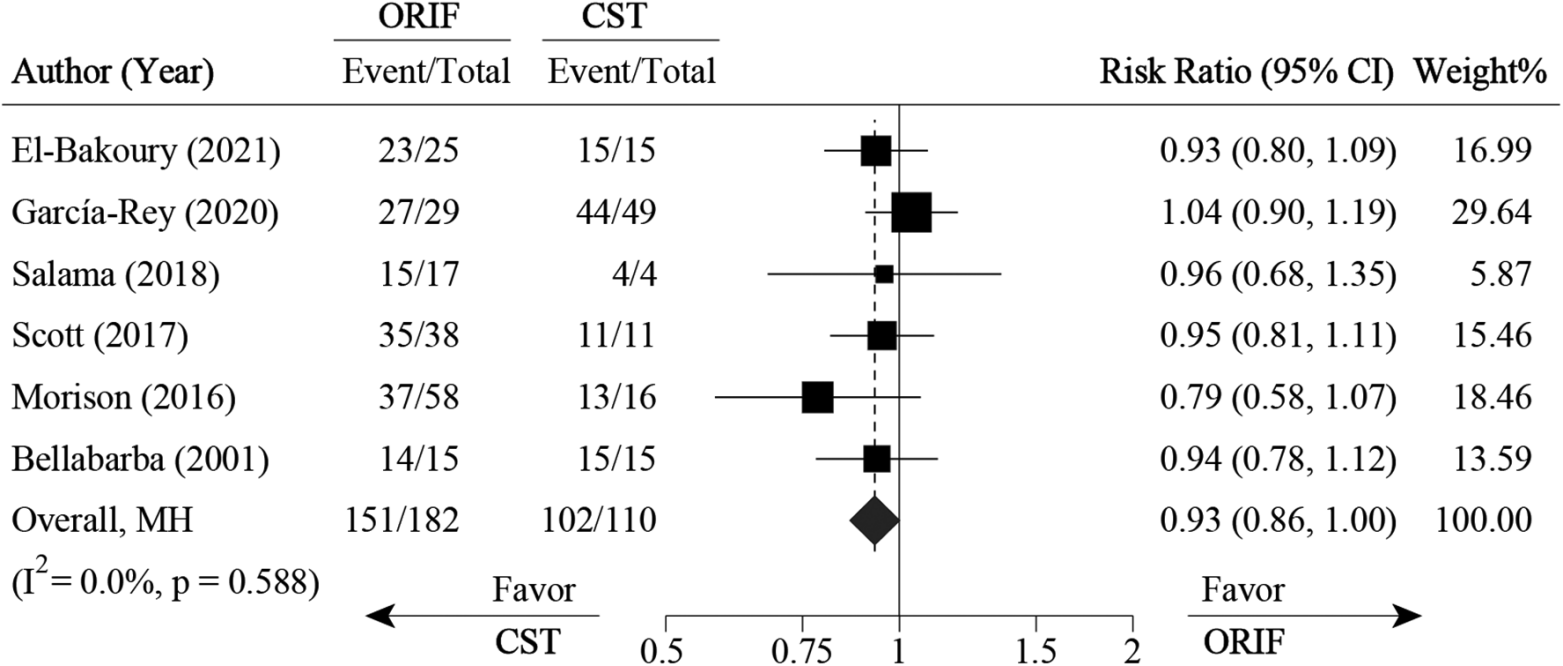
Pooled result for survival rate comparison between ORIF and conservative treatment. CST, conservative treatment or non-surgical treatment; ORIF, open reduction and internal fixation.

**Figure 8:**
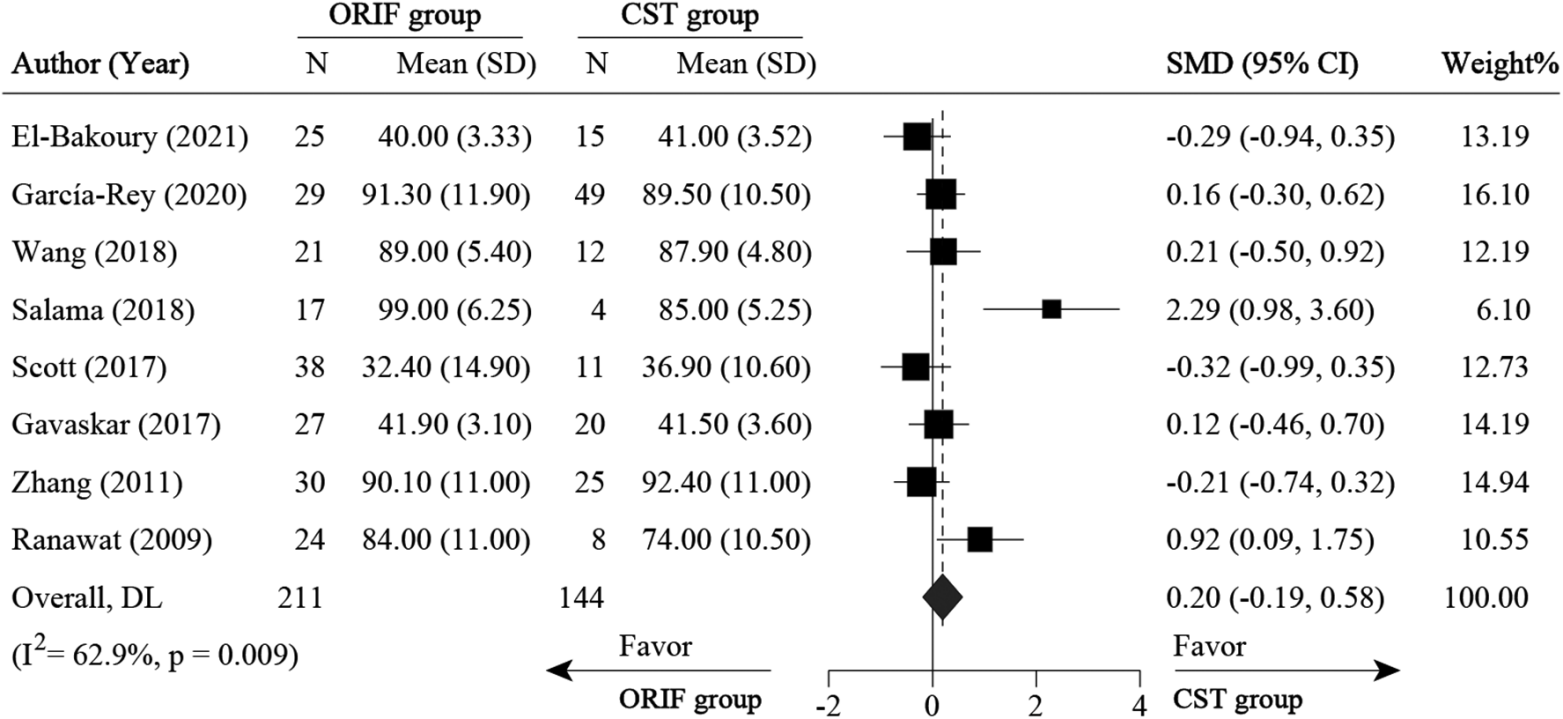
Meta-analysis for HHS and OHS comparison between ORIF and conservative treatment. CST, conservative treatment or non-surgical treatment; HHS, Harris Hip Score; OHS, oxford hip score; ORIF, open reduction and internal fixation; SD, standard deviation; SMD, standard mean difference.

Ten studies (1, 13, 14, 24–27, 32, 35, 37) divided the patients into ORIF and CST groups based on the primary treatment methods after AF. For survival rate, six studies (13, 14, 25, 26, 32, 37) were pooled with RR of 0.93 (0.86 to 1.00, 95% CI), which was in favor of the CST group (*P* < 0.05) (Figure 7). No significant heterogeneity was found across these studies (*I*^2 ^= 0%, *P* = 0.588). For clinical outcomes (HHS and OHS), eight studies (1, 13, 14, 24–27, 35) were pooled with a standardized mean difference (SMD) of 0.20 (−0.19 to 0.58, 95% CI) and with no significant difference between the ORIF and CST groups (Figure 8).

### Posttraumatic osteoarthritis vs. primary osteoarthritis

**Figure 9:**
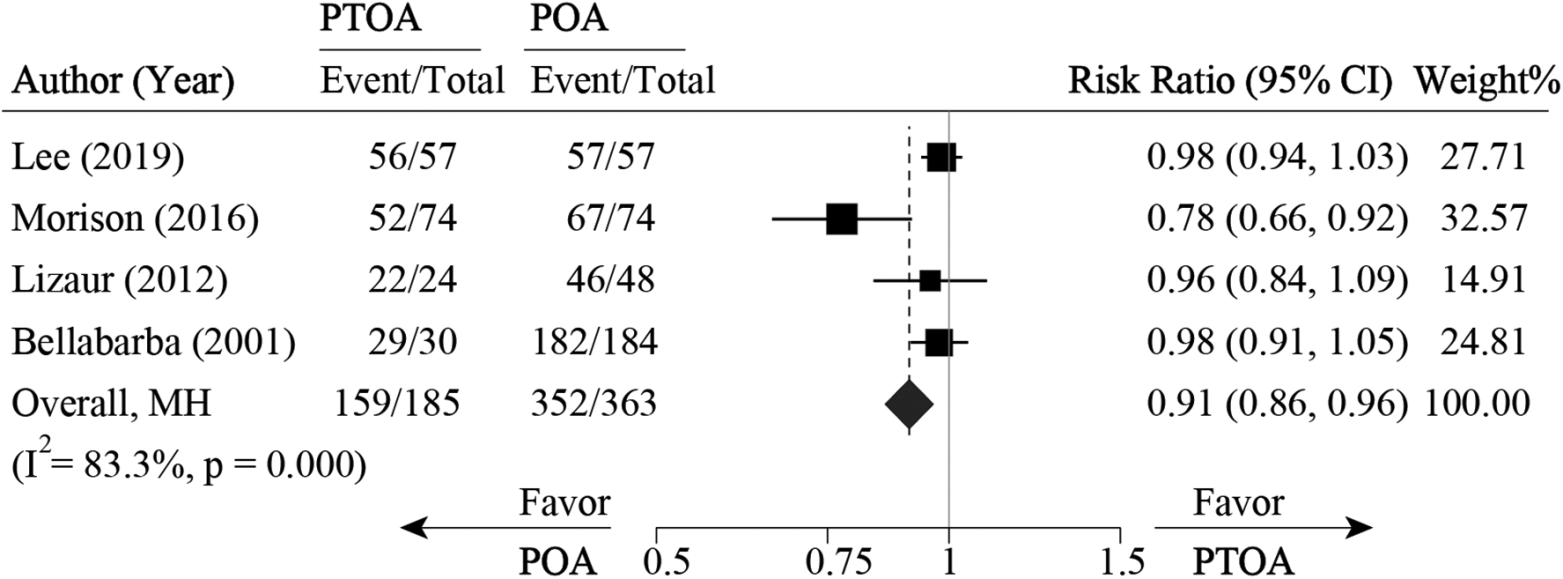
Pooled result for survival rate comparison between PTOA and POA. PTOA, post-traumatic osteoarthritis; POA, primary osteoarthritis.

**Figure 10:**
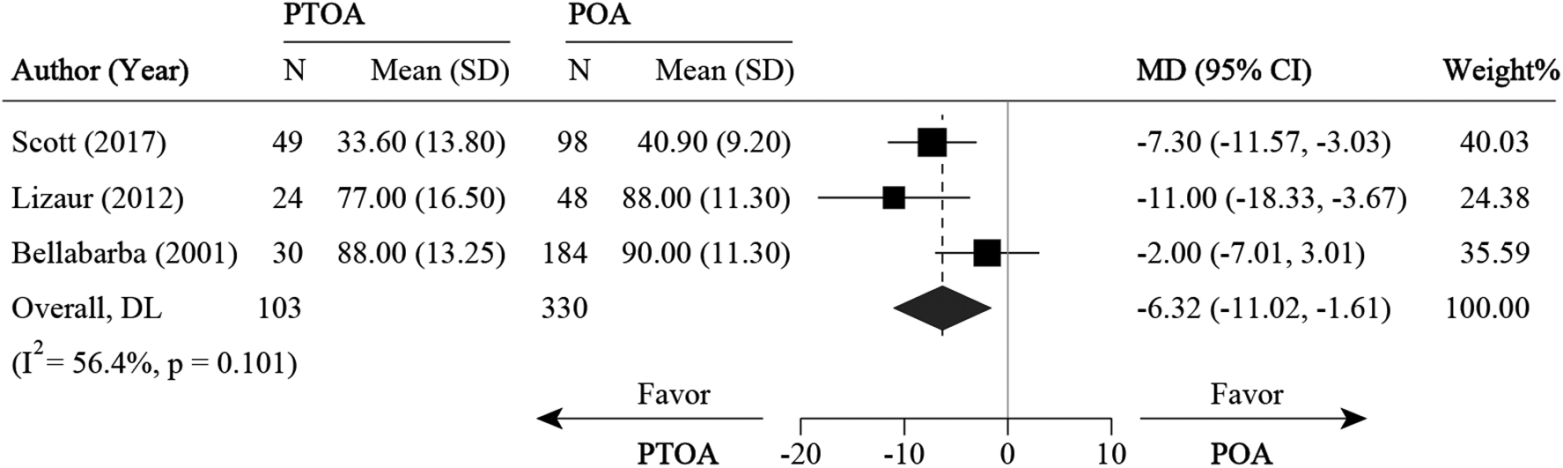
Meta-analysis for HHS and OHS comparison between PTOA and POA. HHS, harris hip score; MD, mean difference; OHS, oxford hip score; PTOA, post-traumatic osteoarthritis; POA, primary osteoarthritis; SD, standard deviation.

Five studies (21, 26, 32, 34, 37) compared PTOA following AF with POA. For survival rate, four studies (21, 26, 32, 34, 37) were pooled with an RR of 0.91 (0.86 to 0.96, 95% CI), which favors the POA group (*P* < 0.05) (Figure 9). For clinical outcomes (HHS), three studies (21, 26, 34, 37) were pooled with an MD of −6.32 (−11.02 to −1.61, 95% CI), which was in favor of the PTOA group (Figure 10). No significant heterogeneity was found among these three studies (*I*^2 ^= 0%, *P* = 0.625).

### Uncemented vs. cemented endoprosthesis

Three studies (28, 30, 38) compared uncemented with cemented THA endoprostheses. For survival rate, three studies were pooled with an RR of 0.89 (0.66–1.18, 95% CI), and no significant difference was observed between the two groups (*P *< 0.05) (Supplementary Figure S6). No study reported clinical outcomes.

### Postoperative complications

The most frequently reported complications across studies were: HO (22 studies), implant dislocation (27 studies), implant loosening (27 studies), and infection (27 studies). The most common clinically signiﬁcant complication was HO, based on the Brooker HO classiﬁcation, which had a median of 9% (IQR 1%–21%) and ranged from 0% to 47%. Rates of implant dislocation were also reported, ranging from 0% to 12%, with a median of 1% (IQR 0%–4%). Rates of implant loosening ranged from 0% to 24%. Rates of infection ranged from 0% to 9%. Iatrogenic nerve injury was the least frequently reported complication at 0%–3%. Revision rates varied: revision surgery rate was reported in 27 studies and ranged from 0%–35%, with a median of 3% (IQR 0%–9%). Table 3 and Supplementary Table S1 present the reported postoperative complications.

**Table 3:** Pooled complications after THA for PTOA following acetabular fracture.

Complication	Event number	Total number	Incidence
Heterotopic ossification	205	910	22.53%
Revision surgery	156	1,137	13.72%
Loosening (acetabular + femur)	100	1,137	8.80%
Dislocation	51	1,095	4.66%
Infection	40	1,162	3.44%
Periprosthetic fracture	14	1,137	1.23%
Nerve injury	12	1,125	1.07%

## Discussion

AFs represent complex injuries of the hip and are associated with high morbidity (32). Restoration of joint congruency plays an important role in therapeutic outcomes. However, PTOA after AF can occur even after anatomical reconstruction (39). Despite modern AF management using improved surgical techniques, the incidence of PTOA is still nearly 30% (40). In such cases, further surgery in the form of THA is recommended to relieve pain and restore function when ORIF fails (39). In the present study, THA was shown to provide adequate symptomatic relief for PTOA secondary to AF.

### Survival rate

There was a wide variation in implant survival rate across the studies included in our analysis, from 57% to 100% at any follow-up time. The overall pooled implant survival rate was 88%. Interestingly, we observed a significant negative correlation between survival rate and follow-up time. At <5 years, the pooled survival rate was 100% (18, 20, 25); however, at ≥15 years, the pooled survival rate decreased markedly to 83%. Two underlying factors might have led to this decrease. First, prosthetic wear or loosening might have played a role. Berry et al. (36) and Roth et al. (30) conducted studies with 20-year follow-ups; survival rates for patients free from acetabular revision for aseptic loosening declined from 87% at 10 years to 71% at 20 years. All revisions after 10 years were performed for wear or loosening. Second, the optimization of prosthesis materials with time might have played a role. Berry et al. (36) found that the use of first- and second-generation uncemented acetabular cups resulted in more polyethylene wear and higher revision rates. Additionally, data from Chiu et al. (33) and Zhang et al. (35) reported 5% and 2% modification rates, respectively, suggesting that cup material and liner choice may affect the durability of THA.

### Clinical outcomes

Comparison between posttreatment and pretreatment was conducted across studies. HHS after THA for PTOA following AF improved in all patients, with 43.25 points postoperatively at the final follow-up. Individual studies included in our meta-analysis showed that the HHS improved, ranging from 27.5 to 60 points (22, 37). Publication bias was observed, as all the studies had positive results. We then conducted a trim-and-fill analysis, and eight studies were filled, which indicated that at least eight studies with negative or null results were not published. Therefore, the clinical effect of THA on HHS improvement for the patients with PTOA following AFs might not be so large.

### Complications

In the current review, HO was the most common complication, with a pooled rate of 22.53%. Patients with previous AF had a higher likelihood of HO (43%) compared with conventional primary THA (16%; 32). HO usually did not require surgical treatment in most patients because symptoms were absent or minimal and well-controlled with CST. Hip dislocation is one of the most common postoperative complications of THA. In our review, the pooled rate of implant dislocation was 4.66%. A recent review reported a dislocation rate of 4.4% after THA following AF as compared with the 0.2%–7% seen in conventional primary THA (39). The pooled rate of infection was 3.44% in the current review. Surgical approaches might play a role in implant infection. Acuña et al. (41) conducted a meta-analysis and found that patients who underwent a direct anterior approach had a significantly reduced risk of infection compared with those who underwent posterior and direct lateral approaches.

### Posttraumatic osteoarthritis vs. primary osteoarthritis

In the current review, five studies compared PTOA following AF with POA, with the pooled implant survival rate favoring POA. When THA patients with PTOA were compared to THA patients with POA, the posttraumatic cases presented more operative challenges and postoperative complications. The inciting trauma considerably altered tissues and anatomical constituents, which presented inherent challenges for surgeons (42). Patients with PTOA showed higher blood loss, transfusion requirements, and operative times for posttraumatic patients as compared to patients with POA (26). Despite these increased surgical challenges, the HHS of patients improved dramatically after undergoing delayed THA. In the present review, for clinical outcomes (i.e., HHS), three studies (26, 34, 37) were pooled with an MD in favor of the PTOA group. The lower postoperative HHS in PTOA patients might be due to the significant impairment of daily function experienced by these patients prior to undergoing THA. Therefore, when making surgical decisions for patients with a history of AF, the risks of suboptimal outcomes and complications of delayed THA should be weighed against the possibility of significant improvements in pain, range of motion, and daily function.

### Open reduction and internal fixation vs. conservative treatment

ORIF or CST was usually the first choice before THA for the treatment of AF. In our review, the pooled survival rate favored CST over ORIF. The underlying factors leading to the pooled survival rate were unclear. Meta-regression was used to further analyze the correlation between AF classification and survival rate; however, there was no significant correlation (*P* = 0.997) (Supplementary Figure S1).

### Limitations

Although our study was conducted in strict accordance with the Cochrane handbook for systematic reviews of interventions (43), there were still some limitations. The main limitation of this study was the relative paucity of high-quality studies; all the trials were case series and retrospective studies, and they lacked control groups. Further research involving randomized-control studies is necessary to reduce the potential for unidentiﬁed confounding relationships. There was considerable variation across the studies in this review; therefore, the clinical signiﬁcance of our study is curtailed by the limited availability of high-quality original data for this unique patient population. In addition, although HHS is an excellent endpoint for assessing THA outcomes, it was not utilized across all the studies, limiting our ability to compare surgical outcomes across different treatment modalities and to draw more robust conclusions. HHS comprises four subscales: pain severity, function, absence of deformity, and range of motion. However, the subscale scores of the HHS were not presented in detail across the included studies, and the scores of pain severity, range of motion, and daily function thus could not be extracted and pooled. HHS can be useful in specific situations but needs to be properly validated before use. Its susceptibility to ceiling effects should be considered. The ceiling effect, also known as the high-limit effect, refers to the phenomenon in which test questions are too easy, so that most individuals generally score higher. Retrospective and non-controlled study designs lead to evidence of a low-quality level. The level of evidence was level III and IV. Future studies should collect and provide more detailed clinical data and analyze the correlations between potential risk factors and clinical efficacy-related indicators to provide more interesting information for readers.

## Conclusion

Despite the difficulties associated with performing THA in patients with PTOA due to AF, THA in patients with PTOA due to AF leads to significant improvement in symptoms and function even at ≥15-year follow-up. Survival rates of implants free from re-operation or revision after THA decreased with follow-up time but could still reach 83% at ≥15 years. THA might be an effective therapeutic method for patients with PTOA due to AF.

## Data Availability

The original contributions presented in the study are included in the article/**Supplementary Material**, further inquiries can be directed to the corresponding author/s.
