# Supplementary material for: Total hip arthroplasty for posttraumatic osteoarthritis secondary to acetabular fracture: An evidence based on 1,284 patients from 1970 to 2018
Source: Front Surg. 2022 Nov 10;9:953976. doi: 10.3389/fsurg.2022.953976 (PMC9684333; doi:10.3389/fsurg.2022.953976)
Supplement: Supplementary file 1 [file Datasheet1.pdf]

Supplementary Material

1 Search Strategy

Step	Keyword
#1	acetabul*
#2	acetabulum
#3	acetabulum fracture
#4	acetabulum fracture
#5	fracture of acetabulum
#6	acetabular fracture
#7	#1 OR #2 OR #3 OR #4 OR #5 OR #6
#8	arthritis
#9	osteoarthritis
#10	post traumatic arthritis
#11	osteoarthrit*
#12	#8 OR #9 OR #10 OR #11
#13	hip replacement
#14	total hip replacement
#15	hip joint replacement
#16	total hip arthroplasty
#17	total hip joint replacement
#18	#13 OR #14 OR #15 OR #16 OR #17
#19	#7 AND #12 AND #18

2 Supplementary Figures and Tables

2.1 Supplementary Figures

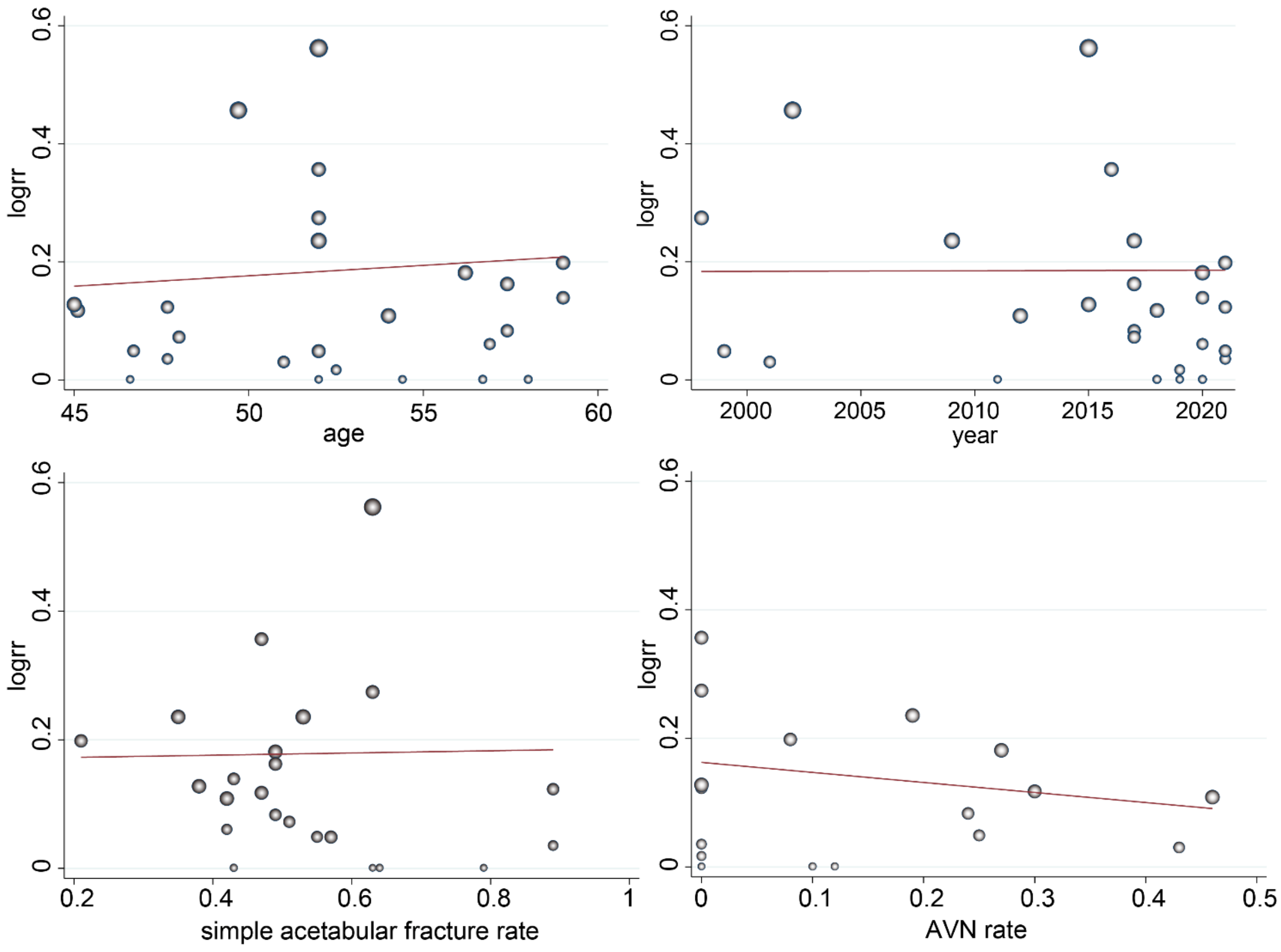

Supplementary Figure 1. Meta-regressions based on age, publication year, simple acetabular fracture rate and AVN rate for implant survival rate. AVN, avascular necrosis of the femoral head. No statistically significant correlations between implant survival rate and age (Coefficient = -0.0007, P=0.948), publication year (Coefficient = -0.0056, P=0.996), simple acetabular fracture rate (Coefficient = 0.0172, P=0.997) and AVN rate (Coefficient = -0.1561, P=0.975), were observed.

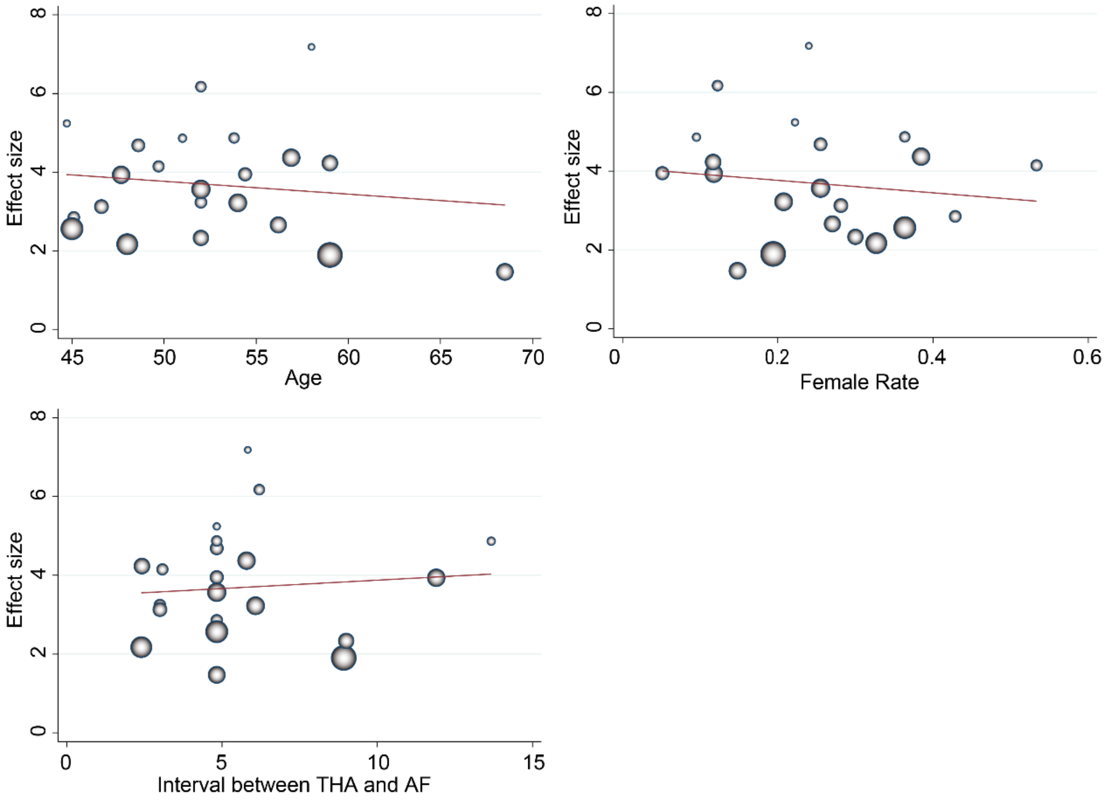

Supplementary Figure 2. Meta-regressions for HHS comparison between posttreatment and pretreatment. HHS, Harris Hip Score. No statistically significant correlations between HHS and age (coefficient = -0.0325, P=0.544), female rate (coefficient = -1.5832, P=0.541, and interval between THA and AF (coefficient = 0.0424, P=0.696), were observed.

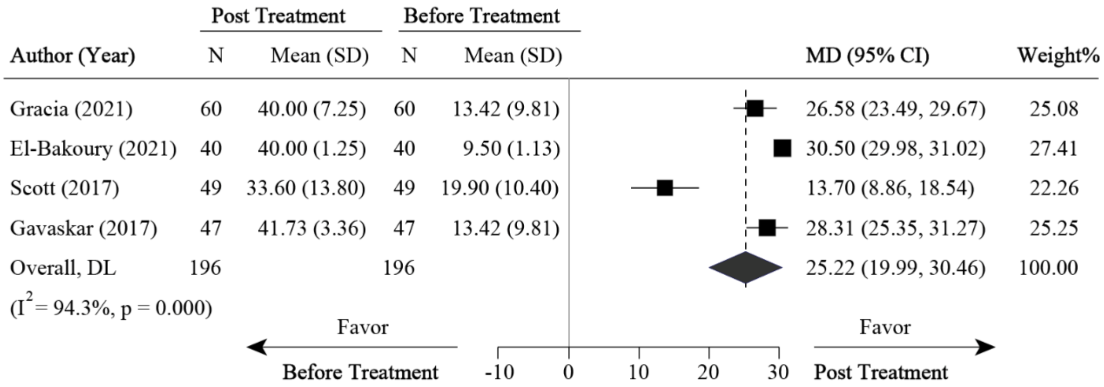

Supplementary Figure 3. Meta-analysis for OHS comparison between posttreatment and pretreatment. MD, mean difference; SD, standard deviation; OHS, Oxford Hip Score.

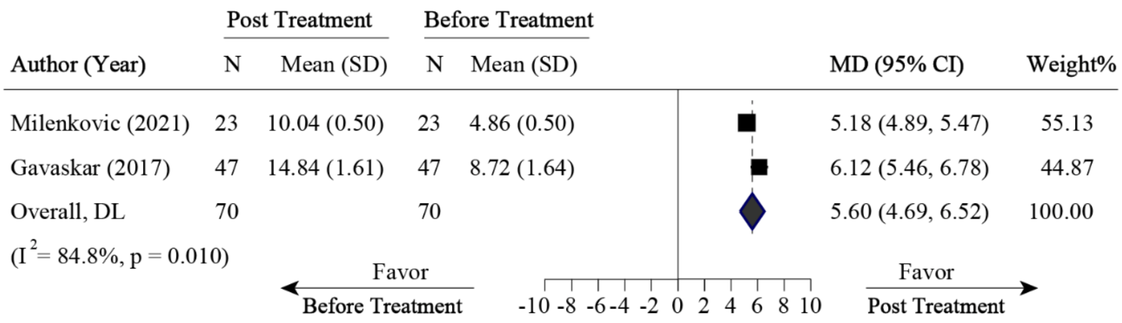

Supplementary Figure 4. Meta-analysis for MDA comparison between posttreatment and pretreatment. MD, mean difference; SD, standard deviation; MDA, Merle d'Aubigne Score.

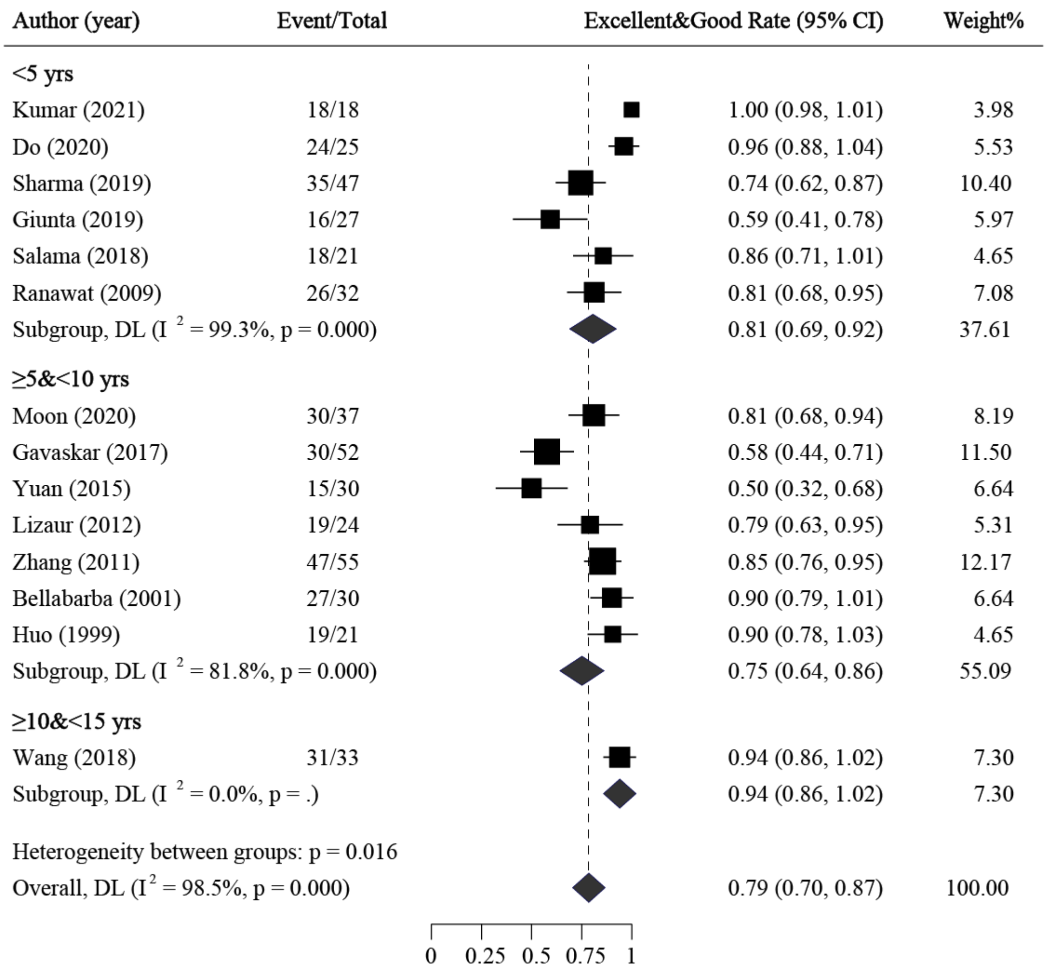

Supplementary Figure 5. Pooled excellent&good rate based on HHS posttreatment. HHS, Harris Hip Score.

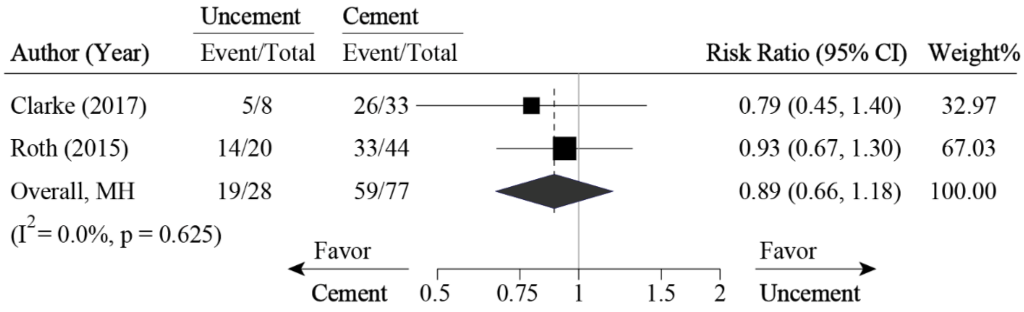

Supplementary Figure 6. Pooled result for survival rate comparison between Uncemented versus cemented endoprosthesis.

2.2 Supplementary Tables

Supplementary Table 1. Complications after THA for PTOA following acetabular fracture.

Author	Year	No of THA patients	Revision surgery	Heterotopic ossification	Dislocation	Loosening (acetabular + femur)	Infection	Periprosthetic fracture	Nerve injury	Implant survival rate
Milenkovic [11]	2021	23	NA	NA	NA	NA	NA	NA	NA	NA
Lucchini [7]	2021	68	8	10	2	2	0	6	0	96.5% at 5 yrs; 88.4% at 10 yrs
Kumar [12]	2021	18	0	1	1	1	1	0	0	NA
Gracia [13]	2021	39	8	3	4	2	4	1	0	82% at 10 yrs
El-Bakoury [14]	2021	40	2	0	2	0	3	0	2	95.2% at 8 yrs
Moon [15]	2020	37	3	14	3	3	1	0	NA	83.4% at 12 yrs
Min [16]	2020	39	0	0	0	0	0	0	0	100% at 8.5 yrs
García-Rey [17]	2020	78	12	NA	0	12	0	0	0	94.1% at 16 yrs
Do [18]	2020	25	0	NA	3	0	0	0	0	100% at 4.2 yrs

Busch [19]	2020	67	8	27	NA	4	0	0	0	87% at 8 yrs
Taheriazam [20]	2019	49	0	0	0	0	0	0	0	100% at 3.7 yrs
Sharma [9]	2019	47	NA	NA	NA	NA	NA	NA	NA	NA
Lee [21]	2019	57	1	0	1	0	0	0	0	98.3% at 10 yrs
Giunta [22]	2019	25	NA	NA	3	NA	4	NA	2	NA
Dawson [23]	2019	25	1	0	1	0	2	0	0	NA
Wang [24]	2018	33	3	10	1	0	1	0	0	88.9% at 17 yrs
Salama [25]	2018	21	0	2	0	0	0	0	0	100% at 1 yr
Scott [26]	2017	49	6	18	3	4	3	1	0	92% at 10 yrs; 85% at 15 yrs
Gavaskar [27]	2017	47	2	17	2	1	1	0	2	93% at 7 yrs
Clarke [28]	2017	44	11	NA	4	2	3	0	0	79% at 7.3 yrs
Morison [32]	2016	74	24	32	8	13	5	1	1	70% at 10 yrs
Yuan [29]	2015	28	3	5	2	0	3	1	0	88% at 5 yrs

Roth [30]	2015	25	12	NA	0	11	0	0	0	57% at 20 yrs
Chiu [33]	2015	NA	NA	NA	NA	NA	NA	NA	NA	NA
Lizaur [34]	2012	24	4	2	0	0	0	0	0	89.7% at 12 yrs
Zhang [35]	2011	49	0	16	1	1	0	0	3	100% at 5 yrs
Ranawat [1]	2009	32	6	14	3	3	6	0	1	79% at 5 yrs
Berry [36]	2002	30	11	NA	3	8	0	2	0	63.33% at 11.6 yrs
Bellabarba [37]	2001	30	1	13	0	2	3	0	0	97% at 10 yrs
Huo [31]	1999	21	1	6	1	4	0	2	1	95.24% at 5.4 yrs
Weber [38]	1998	63	17	15	3	16	0	0	0	76% at 10 yrs, 67% at 15 yrs

NA, not applicable; yrs, years.

Supplementary Table 2. Acetabular fracture patterns according to Letournel's classification system.

Author	Year	AC	AW	PC	PW	T	Simple	AC+PC	AC+PHT	AC+HTS	ACW	PCW	TPW	T-S	Associate
Milenkovic [11]	2021				17	2	19					3	2		5
Lucchini [7]	2021		1	3	35	19	58					3	4		7
Kumar [12]	2021						6								12
Gracia [13]	2021					8	8	14	6			3	7		30
El-Bakoury [14]	2021						22								18
Moon [15]	2020	2		3	10	3	18	5	1			3	7	3	19
Min [16]	2020	1		29			30	3	2			1	2		8
García-Rey [17]	2020	1	2	9	7	8	27	10				12		16	38
Do [18]	2020	1			13	2	16	6	1			1		1	9
Busch [19]	2020	5		5	10	9	29	17	3			2	10	6	38

Taheriazam [20]	2019						NA								NA
Sharma [9]	2019			6	27		33	9						5	14
Lee [21]	2019						NA								NA
Giunta [22]	2019			2	9	2	13	5		5				4	14
Dawson [23]	2019	5		3	4	3	15	6				3	4		13
Wang [24]	2018	2		4	7	3	16	4	3			3	6	2	18
Salama [25]	2018						9								12
Scott [26]	2017	1	1		17	4	23	3	4			4	8	5	24
Gavaskar [27]	2017	4	1	5	9	5	24	4	2			4	4	9	23
Clarke [28]	2017	2	1	2	12	1	18	6	9			2	16	1	34
Morison [32]	2016	2		5	23	5	35	12	4			8	7	8	39
Yuan [29]	2015				6	2	8	1				3	4	5	13

Roth [30]	2015	3		1	27	4	35					11	6	4	21
Chiu [33]	2015						NA								NA
Lizaur [34]	2012	2			8		10					9		5	14
Zhang [35]	2011	1			28	4	33	6					13		19
Ranawat [1]	2009			1	13	3	17	4	1		1	5	3	1	15
Berry [36]	2002						NA								NA
Bellabarba [37]	2001						NA								NA
Huo [31]	1999				12		12	2				5		2	9
Weber [38]	1998	3		1	27	4	35					11	6	4	21
**Total**		32	6	78	294	87	534	117	36	5	1	85	103	77	466
**Percentage**		3.47%	0.65%	8.47%	31.92%	9.45%	47%	12.70%	3.91%	0.54%	0.11%	9.23%	11.18%	8.36%	53%

AC, anterior column; AW, anterior wall; PC, posterior column; PW, posterior wall; T, transverse fracture; AC+PC, anterior column + posterior column; AC+PHT, anterior column + posterior hemitransverse; AC+HTS, anterior column + hemitransverse; ACW, anterior column + anterior wall; PCW, posterior column + posterior wall; TPW, transversal fracture + posterior wall; T-S, transverse-shaped fracture.

Supplementary Table 3. Surgical characteristics for studies which data are available.

**Author**	**Year**	**Operative time (minutes)**	**Blood loss (ml)**	**Hospital stay (days)**	**Blood transfusion (units)**
Lee [21]	2019	145.3± 41.7	795.6 ± 587.8	13.4 ± 8.2	NA
Giunta [22]	2019	110±29, (R 65-170)	827±688.8, (R 152-3019)	23.6±14.1, (R 8-58)	NA
Dawson [23]	2019	91.2	585	5	NA
Wang [24]	2018	188 (R 175 - 321)	800 (400-1700)	NA	NA
Salama [25]	2018	96 (R 55-200)	NA	NA	NA
Gavaskar [27]	2017	86±24 in ORIF, 115±38 in CST, p<0.01	448±105 in ORIF, 652±212 in CST, p<0.01	NA	NA
Roth [31]	2015	170 (R 90-315)	NA	NA	NA
Lizaur [33]	2012	81± 8.8, (R 65-96)	NA	NA	NA
Ranawat [1]	2009	NA	718 (R 100-2000)	NA	NA
Bellabarba [36]	2001	179 (R 90-300)	898 (R 250-2900)	NA	2.2 (R 0-5)

Huo [37]	1999	97 (R 60-190)	960 (R 500-2200)	8 (R 5-13)	NA
Weber [38]	1998	170 (R 90-315)	NA	NA	NA

CST, conservative treatment or non-surgical treatment; NA, not applicable; ORIF, open reduction and internal fixation; R, range.

Supplementary Table 4. Clinical results and conclusions.

Author	Year	Outcome	Comparison result	Conclusion	Journal
Milenkovic [11]	2021	MDA: PRT 4.86(4-6), POT 10.04 (10-12), p<0.001; VAS pain: PRT 9.04, POT 1.95, p<0.001		THA is a method which gives the best results in the treatment of post-traumatic OA of the hip and AVN of the femoral head after previous osteosynthesis of the AF. After THA, life quality and functional status of a patient are significantly improved.	Int Orthop
Lucchini [7]	2021	HHS: PRT 37.6±14.1, POT 88.4±11.6, p<0.001		THAs in post-traumatic OA after AF showed good results despite the fact that specific ceramic-related issues have to be considered.	Arch Orthop Trauma Surg
Kumar [12]	2021	HHS: PRT, POT 89.72±4.24; SF12: PRT, POT 44.29±2.95; Excellent: 11 (61.1%) & Good: 7 (38.9%); Young <40yrs better than old		THA in patients with failed acetabulum fracture fixation surgery provides a reliable option with satisfactory outcomes along with a better quality of life.	J Clin Orthop Trauma
Gracia [13]	2021	HHS: POT 85 (60-90) for delayed, 73 (45-92) for acute, P=0.049. OHS: POT 40 (19-48), 32 (22-44), P=0.011. PMA: POT 16 (10-17), 12 (8-17), P=0.007		Acute THA in the setting of AF is a technically challenging procedure. THA provided satisfactory immediate stability and good survivorship at 10 years in a medically vulnerable patient population.	Eur J Orthop Surg Traumatol
El-Bakoury [14]	2021	OHS: PRT 9.5 (7-11.5), POT 40 (39-44) p<0.001	No difference between both groups regarding acetabular defects, bone graft use and THA outcomes.	Delayed cementless acetabular THA in patients with previous failed AF treatments produces good clinical outcomes with excellent survivorship	Bone Jt Open
Moon [15]	2020	HHS: PRT 42.9 (17-70), POT 83.5 (29-97), p<0.001. Excellent & Good: 30 (81.1%)	Median surgery time and intra-operative blood loss were higher in DM-CHP than those in ORIF. Early medical complication rate was higher for a combined approach as compared with a single posterior approach in DM-CHP (p	THA secondary to an operatively treated AF provides good symptomatic relief, but shows relatively inferior survival rates, and clinical failure was related to post-traumatic arthritis with acetabular non-union.	Archives of orthopaedic and trauma surgery

			= 0.003). Dislocation rate was 7.7% in DM-CHP. Revision rate was higher in ORIF (20% versus 7.7%). HHS was similar in both groups.		
Min [16]	2020	HHS: PRT 44.4, POT 93.1, p<0.001.		Our encouraging results support the continued use of this type of polyethylene in patients after AFs.	Indian J Orthop
García-Rey [17]	2020	HHS: ORIF: PRT 53.1±4.9, POT 91.3±11.9, p<0.001; CST: PRT 50.2±6.4, POT 89.5±10.5, p<0.001; ORIF vs CST p=0.514.	Three acetabular components were revised for aseptic loosening in Group 1 and 2 in Group 2. The survival rate for cup loosening at 16 years was 90.6% (95% confidence interval [CI], 78.1–100) for Group 1 and 94.1% (95% CI, 86.5–100) for Group 2 (p = 0.76). There were 2 sciatic palsies in Group 2 after osteosynthesis. The mean preoperative clinical score and postoperative range of mobility were better in Group 1. There were more heterotopic ossifications in Group 2.	Despite the good results found in both groups, THA after previous osteosynthesis for AFs had more complications than a primary THA in conjunction with acetabular reconstruction.	Hip Int
Do [18]	2020	HHS: PRT 41 (18-47), POT 88 (77-100), p<0.01. Excellent & Good: 24 (96%)		Outcomes of cementless THA after failed internal fixation for AFs were satisfactory. However, a relatively high incidence of postoperative dislocation is still a concern.	J Orthop Surg
Busch [19]	2020	HHS: PRT ±, POT 75.7±21.3, p<0.001		Arthroplasty due to PTOA after AF resulted in decreased overall survival rates and poorer clinical outcome as compared to primary arthroplasty.	Arch Orthop Trauma Surg
Taheriazam [20]	2019	HHS: PRT 47 (31-66), POT 89 (79-95) p<0.001; WOMAC: PRT 15 (7-20), POT 4 (0-11)		The conversion to THA after PTOA in AF can lead to reasonable pain relief and functional improvement.	Orthop Res Rev
Sharma [9]	2019	HHS: Excellent & Good: 35 (74.47%)		HHS and quality of life scores had a tendency to improve over time.	J Clin Orthop Trauma

Lee [21]	2019	UCLA: PRT 3.6±1.3; POT 4.9±1.9	Posttraumatic patients had longer operation time and larger volume of transfusion than osteonecrotic patients. However, medium-term results and survivorship were similar with those of osteonecrotic	Posttraumatic patients had longer operation time and larger volume of transfusion than osteonecrotic patients. Medium-term results and survivorship were similar with those of osteonecrotic patients.	J Orthop Surg
Giunta [22]	2019	HHS: POT 70.4±23.6; PMA: POT 14.3±4.2; Excellent & Good: 16 (64%)	Mean HARRIS score after conservative treatment was 65.6 ± 13.6 (38–90), and mean PMA score was 12 ± 4 (5–18) at last follow-up. The differences were statistically significant in comparison with patients with early THA ($p < 0.05$). Mortality rate was higher in the non-operative group (8/21,38%, $p < 0.05$), while THA group had the highest complication rate with 20 complications in 27 patients (73%) versus 15 complications in 21 patients (56%) with conservatively treatment, $p < 0.05$.	Primary THA for AF in the elderly population might be a good therapeutic option that allows return to the previous daily life activity.	Int Orthop
Dawson [23]	2019	NA		Acceptable post-operative outcomes were demonstrated throughout the case series.	Eur J Orthop Surg Traumatol
Wang [24]	2018	HHS: PRT 44±11.9 (R 27-58), POT 88.6±5.1 (R 74-94); Excellent & Good: 31 (93.9%)	No difference in THA outcomes between the two groups. Surgery duration, blood loss, and transfusion requirement were greater in the ORIF group than in the non-ORIF group ($p < 0.05$). overall survival rate was similar in both groups,	Initial fracture treatment does not influence the outcome of delayed THA, and modern ceramic bearing has promising results in the long-term follow-up.	J Orthop Surg Res
Salama [25]	2018	HHS: PRT 38 (0-70), POT 92 (19-100) $p<0.001$; WOMAC: PRT 63 (42-92), POT 4 (0-19); Excellent & Good: 18 (86%); HHS: ORIF 99 (75-100) vs CST 85 (79-100), $p<0.05$.	HHS was higher in the CST group as compared to ORIF	Cementless THA is an ideal treatment for posttraumatic hip arthritis with anatomic restoration of the hip center to improve the functional results and decrease the incidence of complications and revision rate.	Eur J Orthop Surg Traumatol

Scott [26]	2017	OHS: PRT 19.9±10.4 (R 5-36), POT 33.6±13.8 (R 3-48)	THA complication rates (all complications, heterotopic ossification, leg length discrepancy > 10 mm) were significantly higher following AF compared with atraumatic OA/AVN and OHSs were inferior: one-year OHS (35.7 versus 40.2, p = 0.026); and final follow-up OHS (33.6 versus 40.9, p = 0.008).	Cemented THA is a reasonable option for the sequelae of AF. Higher complication rates and poorer PROMs, compared with patients undergoing THA for atraumatic causes, reflects the complex nature of these cases.	Bone Joint J
Gavaskar [27]	2017	OHS: ORIF: PRT 16.7±2.8, POT 41.9±3.1, p<0.001; CST: PRT 9±3.7, POT 41.5±3.6, p<0.001; ORIF vs CST p=0.68. MDA: ORIF: PRT 9.7±1.2, POT 15.1±1.7, p<0.001; CST: PRT 7.4±2.1, POT 14.5±1.5, p<0.001; ORIF vs CST p=0.20.	THA for a failed AF is greatly facilitated by initial surgical treatment. no difference in THA outcomes between the two groups; higher number of patients with bone defects requiring complex acetabular reconstruction in CST group. Patients in group A returned to work much sooner compared to patients in group B (P= 0.004).	THA for a failed AF is greatly facilitated by initial surgical treatment.	J Arthroplasty
Clarke [28]	2017	Survival 10 yrs: UC 57% vs C 80%; HHS: PRT 53±10, POT 82±16.	Ten-year revision free arthroplasty survival was 79%. Uncemented arthroplasties had a significantly worse 10-year survival of 57%.	THA secondary to an operatively treated AF provides good symptomatic relief.	Injury
Morison [32]	2016	Survival 10 yrs: AF 57% vs POA 90%, p<0.001	The 10-year survivorship after THA was lower in patients with a previous AF than in the matched cohort	Patients with a prior AF had markedly inferior 10-year survivorship and more frequent serious complications when compared with patients undergoing THA for primary osteoarthritis or AVN.	Clin Orthop Relat Res
Yuan [29]	2015	HHS: PRT 39 (R 3-71), POT 82 (R 21-100); Excellent & Good: 15 (54%), Fair 3 (11%), Poor 10 (35%)		The short-term results of the use of porous metal acetabular components in THA for treatment of posttraumatic OA after AF demonstrate low rates of mechanical failure.	Clin Orthop Relat Res
Roth [30]	2015	HHS: PRT 49, POT 80 (R 51-100). Survival 20 yrs: 57% for all, UC 69% vs C 75%, p=0.6;	No difference in survivals between UC and C.	THA after ORIF of an AF was associated with fair implant survivorship at twenty years after surgery.	J Bone Joint Surg Am

Chiu [33]	2015	Survival 10 yrs: Male sex, age younger than 50 years, large acetabular deficiency, and sclerotic changes of the acetabulum were significant factors contributing to the mechanical failure		Male sex, age younger than 50 years, large acetabular deficiency, and sclerotic changes of the acetabulum were significant factors contributing to the mechanical failure of cementless acetabular reconstruction performed for old AFs treated with open reduction and internal fixation. Use of the Trabecular Metal cup seemed able to prolong the endurance of the acetabular component in the subsequent reconstruction.	Orthopedics
Lizaur [34]	2012	HHS: PTOA: PRT 35.1±7.9 (R 22-52), POT 77±16.5 (R 45-94); Excellent 3 (12.5%): Good 16 (66.7%), Fair 1 (4.2%), Poor 4 (16.6%); POA: PRT 37.0±5.8 (R 22-48), POT 88±11.3 (R 38-98); Excellent 31 (64.5%), Good 13 (27%), Fair (2%), Poor 3 (6.5%); PTOA vs POA, p=0.01. Survival at 12 yrs: PTOA 89.7%; POA 95.8%, p=0.70.	No significant difference between the two cohorts was found with regard to postoperative follow-up, perioperative transfusion requirements, reoperations, and acetabular survival. Between both cohorts, there were significant differences with regard to operative time and postoperative Harris score.	Cementless THA is a suitable treatment for PTOA after AF.	J Trauma Acute Care Surg
Zhang [35]	2011	HHS: Total: PRT 49.5 (R 22-78), POT 90.1 (R 56-100); Excellent 36 (%): Good 11 (%), Fair 2 (%), Poor 2 (%); ORIF: PRT 49.5 (R 30-78), POT 90.1 (R 56-100); CST: PRT 54.3 (R 22-76), POT 92.4 (R 56-100); ORIF vs CST, p<0.05. Favor ORIF.	HHS was significant higher in the ORIF group than in the CST group (P<0.05)	The results of acetabular reconstruction are encouraging in these patients	J Arthroplasty
Ranawat [1]	2009	HHS: Total: PRT 28 (R 0-56), POT 82 (R 20-100); Excellent&Good 26 (81%), Fair 2 (%), Poor 4 (%); ORIF: POT 84, Excellent&Good 21 (88%); CST: POT 74, Excellent&Good 5 (63%); ORIF vs CST, p=0.33.	No difference in HHS between the ORIF and CST groups.	Cementless acetabular fixation in THA demonstrate improved results for PTOA following AF.	J Arthroplasty

Berry [36]	2002	NA		Uncemented sockets had a low rate of loosening in this challenging patient population	Clin Orthop Relat Res
Bellabarba [37]	2001	HHS: PTOA: PRT 41 (R 19-55), POT 88 (R 47-100); Excellent&Good 27 (90%), Fair 1 (3%), Poor 2 (7%); ORIF vs CST, P>0.05: POA: PRT 52, POT 90, Excellent&Good 83%; PTOA vs POA, p=0.10; ORIF vs CST, p=0.33. Survival at 10 yrs: PTOA 97%, POA 99%, p<0.05; ORIF 93%, CST 100%, p<0.05	Ten-year survival rate with revision favors POA as compared PTOA, and favors CST as compared ORIF.	The intermediate-term clinical results of total hip arthroplasty with cementless acetabular reconstruction for PTOA after AF were similar to those after the same procedure for nontraumatic arthritis.	J Bone Joint Surg Am
Huo [31]	1999	HHS: PRT 30, POT 90; Excellent 16 (76.19%), Good 3 (14.29%), Fair 0 (%), Poor 1 (%)		Slightly better than those reported previously in hip replacements done with cement at comparable medium-term follow-up.	J Arthroplasty
Weber [38]	1998	HHS: PRT 49, POT 93, p<0.0001;		THA is a method which gives the best results in the treatment of post-traumatic OA of the hip and AVN of the femoral head after previous osteosynthesis of the AF.	J Bone Joint Surg Am

AVN, avascular necrosis of the femoral head; PRT, pretreatment; CHP, combined hip procedure (ORIF + THA); CST, conservative treatment or non-surgical treatment; POT, post treatment; CoC, ceramic-on-ceramic bearings; UC, uncemented; C, cemented; H, hybrid; THA, total hip arthroplasty; ORIF, open reduction and internal fixation; OREF, open reduction and external fixation; OHS, Oxford hip score; HHS, Harris hip score; MDA, Postel Merle d’Aubigne score; NA, not applicable; PTOA, post-traumatic osteoarthritis; POA, primary osteoarthritis.

h
